# Travel-Time Disparities in Access to Proton Beam Therapy for Cancer Treatment

**DOI:** 10.1001/jamanetworkopen.2024.10670

**Published:** 2024-05-17

**Authors:** Todd Burus, Alexander D. VanHelene, Michael K. Rooney, Krystle A. Lang Kuhs, W. Jay Christian, Christopher McNair, Sanjay Mishra, Arnold C. Paulino, Grace L. Smith, Steven J. Frank, Jeremy L. Warner

**Affiliations:** 1 Markey Cancer Center, University of Kentucky, Lexington; 2 Lifespan Cancer Institute, Rhode Island Hospital, Providence; 3 Department of Radiation Oncology, University of Texas MD Anderson Cancer Center, Houston; 4 Department of Epidemiology & Environmental Health, College of Public Health, University of Kentucky, Lexington; 5 Department of Cancer Biology, Sidney Kimmel Cancer Center, Thomas Jefferson University, Philadelphia, Pennsylvania; 6 Center for Clinical Cancer Informatics and Data Science, Legorreta Cancer Center, Brown University, Providence, Rhode Island

## Abstract

**Importance:**

Proton beam therapy is an emerging radiotherapy treatment for patients with cancer that may produce similar outcomes as traditional photon-based therapy for many cancers while delivering lower amounts of toxic radiation to surrounding tissue. Geographic proximity to a proton facility is a critical component of ensuring equitable access both for indicated diagnoses and ongoing clinical trials.

**Objective:**

To characterize the distribution of proton facilities in the US, quantify drive-time access for the population, and investigate the likelihood of long commutes for certain population subgroups.

**Design, Setting, and Participants:**

This population-based cross-sectional study analyzed travel times to proton facilities in the US. Census tract variables in the contiguous US were measured between January 1, 2017, and December 31, 2021. Statistical analysis was performed from September to November 2023.

**Exposures:**

Drive time in minutes to nearest proton facility. Population totals and prevalence of specific factors measured from the American Community Survey: age; race and ethnicity; insurance, disability, and income status; vehicle availability; broadband access; and urbanicity.

**Main Outcomes and Measures:**

Poor access to proton facilities was defined as having a drive-time commute of at least 4 hours to the nearest location. Median drive time and percentage of population with poor access were calculated for the entire population and by population subgroups. Univariable and multivariable odds of poor access were also calculated for certain population subgroups.

**Results:**

Geographic access was considered for 327 536 032 residents of the contiguous US (60 594 624 [18.5%] Hispanic, 17 974 186 [5.5%] non-Hispanic Asian, 40 146 994 [12.3%] non-Hispanic Black, and 195 265 639 [59.6%] non-Hispanic White; 282 031 819 [86.1%] resided in urban counties). The median (IQR) drive time to the nearest proton facility was 96.1 (39.6-195.3) minutes; 119.8 million US residents (36.6%) lived within a 1-hour drive of the nearest proton facility, and 53.6 million (16.4%) required a commute of at least 4 hours. Persons identifying as non-Hispanic White had the longest median (IQR) commute time at 109.8 (48.0-197.6) minutes. Multivariable analysis identified rurality (odds ratio [OR], 2.45 [95% CI, 2.27-2.64]), age 65 years or older (OR, 1.09 [95% CI, 1.06-1.11]), and living below the federal poverty line (OR, 1.22 [1.20-1.25]) as factors associated with commute times of at least 4 hours.

**Conclusions and Relevance:**

This cross-sectional study of drive-time access to proton beam therapy found that disparities in access existed among certain populations in the US. These results suggest that such disparities present a barrier to an emerging technology in cancer treatment and inhibit equitable access to ongoing clinical trials.

## Key Points

**Question:**

Do certain US populations have inequitable drive-time access to proton beam therapy for cancer treatment?

**Findings:**

In this population-based cross-sectional study of 327 536 032 residents of the contiguous US, individuals aged 65 years and older, living below the federal poverty line, and residing in suburban and rural areas were at greatest risk of having long commutes (≥4 hours) to proton beam therapy for cancer treatment.

**Meaning:**

The current geographic distribution of proton beam therapy facilities in the US is associated with inequitable access to proton beam therapy as a cancer treatment option and may hinder enrollment in ongoing clinical trials.

## Teaser

This cross-sectional study characterizes driving time to facilities providing proton beam therapy for cancer treatment among the US population by demographic characteristics and social determinants of health.

## Introduction

Compared with photon-based radiotherapy, proton beam therapy (PBT) may have an improved toxicity profile due to decreased dose to organs at risk without compromising target coverage.^1^ High upfront levels of investment in equipment and facilities have led to uneven distribution of proton therapy facilities in the United States.^2^ Clinical indications and insurance reimbursement for PBT are increasing but access remains difficult for many individuals given the limited number of available treatment facilities.^3^ Furthermore, patients and families are often required to travel daily for several weeks during treatment, thus compounding the importance of geographic barriers to access. In order to encourage provision of equitable care among diverse populations, it is critical to identify at-risk groups that face travel-based barriers to care. Such knowledge could further inform policy to improve clinical outcomes and participation in clinical trials aimed at evaluating the efficacy of PBT across a myriad of clinical scenarios.

Geographic accessibility, in particular drive time and driving distance, is associated with patient utilization of both proton and photon radiotherapy.^4,5^ A prior investigation by Mallie et al^6^ in 2021 described variation in PBT drive-time accessibility among 36 facilities, with a focus on pediatric and adult populations and disparities at the state and regional level. However, that analysis was largely descriptive in nature without an attempt to address potential confounding between causative variables to explain disparities observed. To our knowledge, a study investigating the geographic accessibility of proton therapy facilities that considers social determinants of health (SDOH) has yet to be performed.

In this study, we examined the drive-time accessibility of all full-service proton facilities operational in the US as of September 2023. Furthermore, we sought to uncover multivariable associations among these factors that would better describe accessibility issues than an unadjusted approach.

## Methods

This cross-sectional study was deemed exempt from review and informed consent was waived by the University of Kentucky institutional review board because we used only publicly available deidentified data, in accordance with 45 CFR §46. We followed the Strengthening the Reporting of Observational Studies in Epidemiology (STROBE) reporting guideline.

### Data Sources

We compiled a list of 42 PBT facilities listed as “currently operating” by the National Association for Proton Therapy on September 1, 2023. We excluded 2 facilities that primarily treat children (St Jude Children’s Research Hospital and Cincinnati Children’s Proton Therapy Center). Geocoding in ArcGIS Business Analyst Pro (ESRI ArcGIS Pro 3.1) provided coordinates for all included PBT facility addresses (eTable 1 in Supplement 1).

We collected census tract population estimates from the US Census Bureau’s 5-Year (2017-2021) American Community Survey estimates using the tidycensus package version 1.4.1 in the R software environment version 4.2.3.^7,8^ The American Community Survey is a survey of population characteristics collected from a nationwide sample of approximately 3.5 million households annually. Along with total population, we included counts and percentage of the total population by census tract for the following population subgroups: Hispanic (all races), non-Hispanic Asian, non-Hispanic Black, non-Hispanic White, aged 65 years or older, living below the federal poverty line, unemployed, uninsured, and living with a disability. We also collected count and percentage of households by census tract with limited vehicle availability (1 or fewer available vehicles) and no broadband internet access. Race and ethnicity variables were classified in accord with methods used in the 2017 to 2021 American Community Survey.^9^

Since Rural-Urban Commuting Area codes were not available for tracts defined by the 2020 Census at the time of analysis, we denoted census tract urbanicity using the 2013 Rural-Urban Continuum Code (RUCC).^10^ Tracts were categorized as urban (RUCC 1-3), suburban (RUCC 4-5), and rural (RUCC 6-9) based on the containing county.

To assess drive times for potential clinical trials, the precise locations for 63 National Cancer Institute (NCI)–designated Cancer Centers were obtained from the NCI GIS Portal for Cancer Research. Seven basic laboratory centers were excluded, as were St Jude Children’s Research Hospital (serves only pediatric patients with cancer) and the University of Hawaii Cancer Center (not in the contiguous US).

### Statistical Analysis

We calculated traffic-aware driving times from the land-based geographic centroid of every census tract in the contiguous US to the nearest PBT facility using the ArcGIS US 2023 Business Analyst dataset with network routes based on contemporaneous roads and average traffic patterns. Driving times were unavailable for 49 tracts due to their centroid being located in a geographically remote area without road access. We imputed driving times for these tracts using a k-nearest neighbor mean of the driving times for the 5 nearest census tracts. We measured driving times in minutes on a continuous scale and calculated 2 categorical driving-time variables: driving times of 0 to less than 1 hour, 1 hour to less than 2 hours, 2 hours to less than 3 hours, 3 hours to less than 4 hours, and 4 or more hours; and a dichotomous indicator for very long commutes and/or poor vehicle access (driving time of 4 or more hours).

We assigned the driving time from the centroid of a census tract to all population totals for that tract and calculated (weighted) median driving times and corresponding IQRs. We stratified census tracts into quintiles based on increasing population percentage for demographic and SDOH factors and compared driving-time distributions. Because analyses of driving times apply to the entire population of the contiguous US, it was not appropriate to perform tests of statistical significance.

We built binary logistic regression models using the dichotomous driving-time variable to identify SDOH factors associated with poor vehicle access to PBT facilities.^11^ First, we performed univariable analyses, and then constructed a full model using all statistically significant univariable factors. We applied backward stepwise selection with Bayesian Information Criteria to arrive at a final multivariable model. The final set of covariates considered for logistic regression analysis included percentages of the population that were (1) aged 65 years or older, (2) uninsured, (3) living below the federal poverty line, and (4) living with a disability; along with percentages of households with (5) limited vehicle availability and (6) no broadband access (all measured in increments of 10 percentage points). We added urbanicity to the final multivariable model to account for its association with SDOH factors. All statistical tests performed were 2-sided with a *P* < .05 significance level; odds ratios (ORs) and 95% CIs are reported. We used the ResourceSelection version 0.3-6 and car version 3.1-2 packages in R version 4.2.3 (R Project for Statistical Computing) to calculate goodness of fit and variance inflation factors (VIFs) for all models. All statistical analyses were performed from September to November 2023.^12,13^

To visualize spatial patterns in SDOH variables and driving times to the nearest PBT facility, we constructed bivariate choropleth maps using ArcGIS Pro. First, we identified spatial clusters of high and low values for the statistically significant SDOH variables (percentage of population age ≥65 years, living below federal poverty line) by calculating the Getis-Ord Gi* statistic for each census tract. We used fixed distance bands of 25 miles and applied the false discovery rate correction. We then mapped the Getis-Ord Gi* statistics for each SDOH variable against driving time to the nearest PBT facility. We used a 3 × 3 legend, with cluster *z*-scores split at −2.576 and +2.576 and driving times split at 2 and 4 hours. We included currently operating PBT facilities and NCI-designated Cancer Centers as additional features in these maps.

## Results

We analyzed PBT access for a population of 327 536 032 residents of the contiguous US; 60 594 624 (18.5%) were Hispanic, 17 974 186 (5.5%) were non-Hispanic Asian, 40 146 994 (12.3%) were non-Hispanic Black, and 195 265 639 (59.6%) were non-Hispanic White persons; and 282 031 819 (86.1%) resided in urban counties.

### Census Tract Driving Times

**Figure 1.**
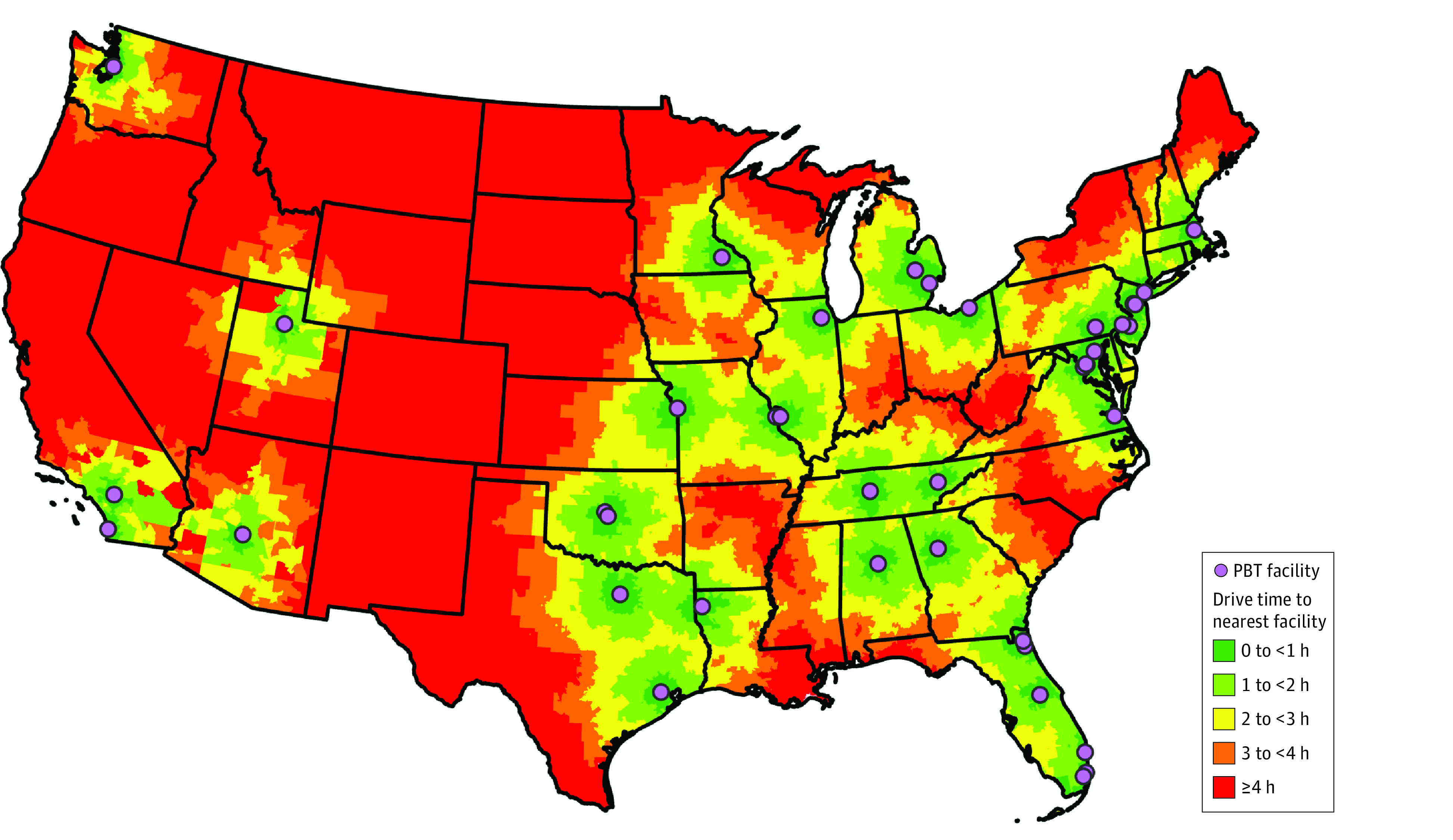
Driving Time to Nearest Proton Beam Therapy (PBT) Facility From Census Tract Land-Based Geographic Centroid^a^ ^a^The geographic centroid of a census tract is the geometric center of the geographic region defined by the census tract boundaries. If the geometric center lies outside of the census tract or in a body of water, the centroid is given as the nearest point on land within the census tract closest to the geometric center.

Median (IQR) driving time to the nearest PBT facility across all census tracts in the contiguous US (n = 83 548) was 102.5 (41.2-198.3) minutes with a maximum driving time of 777.4 minutes (Census Tract 9406, Valley County, Montana). Figure 1 displays a map of all currently operating PBT facilities with census tract driving times categorized by hours from the nearest facility.

### Population Driving Times

Accounting for population distribution, the weighted median (IQR) driving time to the nearest PBT facility for individuals living in the contiguous US was 96.1 (39.6-195.3) minutes. There were 119.8 million US residents (36.6%) who lived within a 1-hour drive of the nearest proton facility, and 53.6 million (16.4%) required a commute of at least 4 hours (Table 1).

**Table 1.** Population Access to Proton Beam Therapy Facilities by Driving Time Categories in the United States

Driving time to proton beam therapy facility, h	Population, millions, No. (%)
0 to <1	119.8 (36.6)
1 to <2	67.7 (20.7)
2 to <3	45.3 (13.8)
3 to <4	41.1 (12.6)
≥4	53.6 (16.4)

Table 2 displays median driving times and percentage of population (or households) with poor vehicle access (≥4 hours away) to PBT facilities for various population subgroups. Median (IQR) driving times for non-Hispanic White (109.8 [48.0-197.6] minutes), those aged 65 years or older (103.0 [43.6-196.7] minutes), those with a disability (107.7 [44.8-201.1] minutes), those living below the federal poverty line (103.6 [40.6-201.1] minutes), households with no broadband access (114.4 [47.0-202.1] minutes), and those living in suburban (164.8 [109.3-229.9] minutes) and rural (190.3 [143.9-276.1] minutes) areas were numerically higher than the population median. A disproportionate amount of the populations of non-Hispanic Asian (12.2 million [20.0%]), aged 65 years and older (8.7 million [16.6%]), persons living with a disability (6.9 million [17.0%]), persons living below the federal poverty line (7.0 million [17.2%]), households with no broadband access (2.7 million [16.8%]), and persons living in suburban (7.8 million [22.3%]) and rural (3.5 million [33.2%]) areas experienced poor access. Non-Hispanic Asian people had the shortest median (IQR) driving time (61.0 [30.0-194.2] minutes) to the nearest PBT facility among subgroups explored, whereas non-Hispanic Black had the smallest population percentage with poor access (4.0 million [9.9%]).

**Table 2.** Comparison of Access to Proton Beam Therapy Facilities by Population Subgroups in the United States, 2017-2021^a^

Category	Total, millions, No. (%)	Driving time, median (IQR), min^b^	Poor access to proton beam therapy facilities, millions, No. (%)^c^
Population	327.5 (100)	96.1 (39.6-195.3)	53.6 (16.4)
Race and ethnicity			
Hispanic (all race)	60.6 (18.5)	76.9 (33.1-201.1)	12.2 (20.2)
Non-Hispanic Asian	18.0 (5.5)	61.0 (30.0-194.2)	3.6 (20.0)
Non-Hispanic Black	40.1 (12.3)	63.3 (29.5-172.2)	4.0 (9.9)
Non-Hispanic White	195.3 (59.6)	109.8 (48.0-197.6)	31.0 (15.9)
Social determinants of health			
Age ≥65 y	52.5 (16.0)	103.0 (43.6-196.7)	8.7 (16.6)
Uninsured	28.3 (8.7)	93.1 (35.9-195.0)	4.6 (16.4)
With disability	40.8 (12.5)	107.7 (44.8-201.1)	6.9 (17.0)
Living below federal poverty line	40.5 (12.4)	103.6 (40.6-201.1)	7.0 (17.2)
Urbanicity			
Suburban	35.1 (10.7)	164.8 (109.3-229.9)	7.8 (22.3)
Rural	10.4 (3.2)	190.3 (143.9-276.1)	3.5 (33.2)
Households	123.3 (100)	98.5 (39.7-195.3)	20.0 (16.2)
Social determinants of health			
Limited vehicle availability	50.3 (40.8)	90.9 (34.6-187.6)	7.4 (14.7)
No broadband	16.0 (13.0)	114.4 (47.0-202.1)	2.7 (16.8)

^a^ Data are from the American Community Survey 5-Year Estimates, 2017 to 2021.

^b^ Population and population subgroup driving times calculated by assigning the applicable number of individuals as weights to their corresponding census tract driving times and computing the weighted medians and interquartile ranges.

^c^ Poor access to PBT facilities is defined as a 1-way commute of greater than or equal to 4 hours to the nearest facility.

Analyzing census tract driving-time distributions by quintiles for racial and ethnic subgroups, we found that median driving times increased with increasing quintiles of non-Hispanic White population and decreased with increasing quintiles of Hispanic, non-Hispanic Asian, and non-Hispanic Black populations (eFigure in Supplement 1). Likewise, median driving times increased with increasing quintiles of population age 65 years or older, living with a disability and with no household broadband access (eFigure in Supplement 1). Median drives times generally increased for increasing quintiles of living below the federal poverty line, with the exception of a slight decrease in the last quintile. Populations uninsured and with limited vehicle availability were found to have nonlinear associations between median driving times and increasing quintiles, with the last quintile of households with limited vehicle availability experiencing the shortest median driving time.

### Modeling Poor Vehicle Access

Table 3 summarizes univariable and multivariable logistic regressions to identify census tract–level covariates associated with poor vehicle access to PBT facilities. The percentage of population uninsured was not significant under univariable analysis and was therefore excluded from the full multivariable model. Percentage with no broadband access was not significant in the full model. Backward selection removed population with no broadband access and population living with a disability. The remaining variables were retained and significant in the final multivariable model. Under this model, the odds of poor vehicle access to PBT facilities increased for each 10% increase in population aged 65 years or older (OR, 1.09 [95% CI, 1.06-1.11]) and living below the federal poverty line (OR, 1.22 [95% CI, 1.20-1.25]), and decreased for each 10% increase in population with limited vehicle availability (OR, 0.83 [95% CI, 0.82-0.84]). Compared with urban census tracts, suburban census tracts had a 40.6% increase (OR, 1.41 [95% CI, 1.33-1.48]) and rural census tracts had a 144.5% increase (OR, 2.45 [95% CI, 2.27-2.64]) in odds of poor access to PBT facilities. The final multivariable model demonstrated goodness of fit via the Hosmer and Lemeshow test (*P* = .23) and all independent variables had VIF less than 2.

**Table 3.** Logistic Regression Analyses to Identify Covariates Associated With a 4-Hour Drive or Longer to Nearest Proton Beam Therapy Facility

Characteristic	Logistic regression analyses, OR (95% CI)	
	Univariable^a^	Multivariable^b^
Age ≥65 y	1.08 (1.06-1.10)	1.09 (1.06-1.11)
Uninsured	1.01 (0.99-1.04)	NA
With disability	1.12 (1.13-1.20)	NA
Limited vehicle availability	0.88 (0.87-0.89)	0.83 (0.82-0.84)
Living below federal poverty line	1.04 (1.03-1.06)	1.22 (1.20-1.25)
No broadband	1.08 (1.06-1.10)	NA
Metropolitan status		
Urban	1 [Reference]	1 [Reference]
Suburban	1.66 (1.57-1.74)	1.41 (1.33-1.48)
Rural	2.94 (2.74-3.16)	2.45 (2.27-2.64)

Abbreviations: NA, not applicable (not in final model); OR, odds ratio.

^a^ ORs are given per quintile increase in variable of interest.

^b^ Multivariable model adjusted for age, vehicle availability, poverty status, and urbanicity.

### Visualizing Factors Associated With Poor Vehicle Access

**Figure 2.**
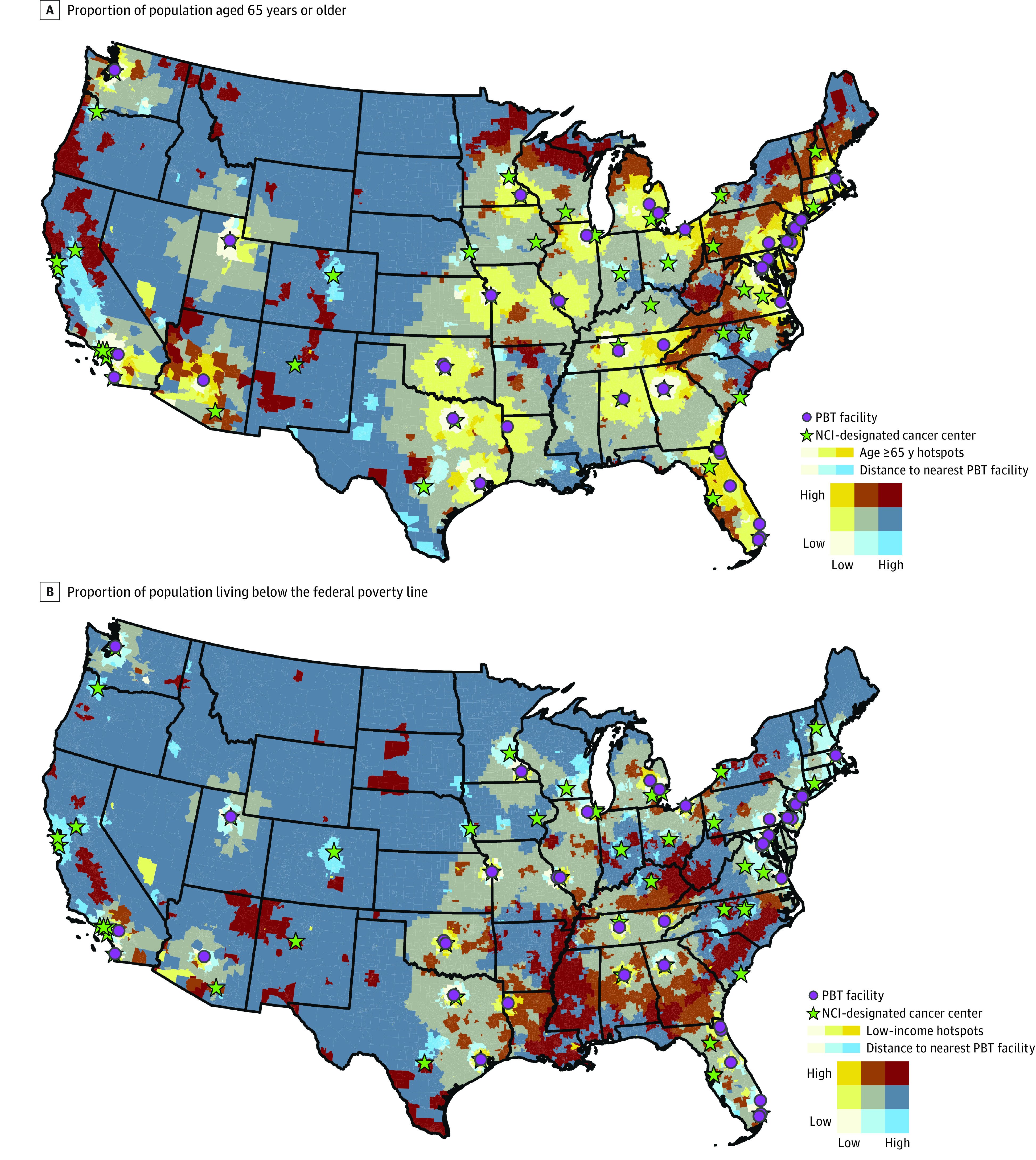
Bivariate Maps of Social Determinants of Health Variables and Driving Time to Nearest Proton Beam Therapy (PBT) Facility The figure shows the geographic association between driving time to the nearest PBT facility and (A) the proportion of population aged 65 years or older and (B) living below the federal poverty line (low income). Social determinants of health variables, age and income status, are displayed as less than −2.576 Gi* statistic (low), between −2.576 and +2.576 (middle), and greater than 2.576 (high). Driving time is displayed as less than 2 hours (low), at least 2 and less than 4 hours (middle), and at least 4 hours (high).

Bivariate maps demonstrate the spatial association between driving times to the nearest PBT facility and the 2 SDOH variables with significantly increased odds of poor vehicle access in the final multivariable model (Figure 2). In particular, the bright red areas (high-high) indicate census tracts that were at least 4 hours from the nearest PBT facility and in a statistically significant hotspot for percentage of the population aged 65 years or older (or, alternatively, population living below the federal poverty line).

### Driving Times to NCI-Designated Cancer Centers

Of the 63 NCI-designated cancer centers analyzed, 23 were run or affiliated with existing PBT facilities, with a median (IQR) driving time between locations of 4.6 (1.8-13.7) minutes. The remaining 40 centers had a median (IQR) driving time to the nearest PBT facility of 132.5 (60.6-199.1 minutes). Six cancer centers were located at least 4 hours from the nearest PBT facility (Figure 2; eTables 2-3 in Supplement 1).

## Discussion

In this investigation, we identified extreme heterogeneity in drive-time accessibility to PBT facilities across the US, with only 36.6% of the population living within 1 hour of a currently operating facility, and 16.4% at a driving distance of 4 or more hours. Furthermore, we identified significant demographic and SDOH factors associated with PBT access, highlighting current disparities to receiving PBT, either for routine clinical indications or as part of a clinical trial. Patients who are older (aged ≥65 years), living below the poverty line, and living in suburban or rural areas, were at highest risk of geographic isolation from PBT facilities. Non-Hispanic White individuals tended to have longer drive-times, likely related to higher population percentages in suburban and rural regions. Drive-time accessibility presents a substantial barrier to the delivery of PBT at scale for the US population today. While temporary overnight accommodations near a proton therapy facility are an option, most people with cancer prefer to receive their treatments in a location that allows for continued residence in the home environment. Furthermore, overnight accommodations are often not covered by insurance and become an additional source of financial burden associated with cancer treatment.^14,15^ These results have broad and important ramifications for patients, radiation oncology clinicians, researchers, cancer center administrators, and policy makers. Being aware of drive-time requirements may also improve shared decision making for clinicians and patients when considering PBT as a potential treatment option.

Maillie et al^6^ published a brief report in 2021 describing PBT accessibility for the 36 facilities operational in the US at that time, with a specific focus on differences between adult and pediatric populations.^6^ Median drive times for pediatric (1.61 hours) and adult (1.64 hours) populations were reported. They additionally reported findings by state and US region, showing large variation across these levels. Our investigation confirms and builds upon this previous work, using an updated list of PBT facilities and a more in-depth analysis of population demographics and SDOH variables.

The receipt of radiotherapy (either proton- or photon-based) presents unique travel-related challenges for patients compared with other oncologic treatment modalities. The effects of increased travel time on oncologic surgery and systemic therapies are mostly relegated to the acute phases of diagnosis and/or treatment, when the need for specialty care tied to a specific location is highest. In contrast, a course of radiotherapy typically includes several weeks of daily outpatient treatment at a specialized facility. Previous studies have demonstrated the negative effect of increased travel distance on the use of indicated radiotherapy treatment for specific cancers.^16,17,18^ Our findings that individuals aged 65 years and older and those living below the federal poverty line are more likely to experience poor access to PBT specifically further complicates matters. Research shows that older patients are already less likely to be prescribed radiotherapy independent of travel considerations, and the disruption caused by radiotherapy regimens is often accompanied by substantial financial burdens (eg, lost wages, travel expenses, cost of temporary relocation) which may inhibit use among patients with low income.^19,20,21,22,23,24^

Understanding these barriers is also important with respect to future research on PBT. At the time of this writing, there are several indications for PBT supported by the National Comprehensive Cancer Network (NCCN) guidelines, including select pediatric tumors, thymic tumors, uveal melanomas, esophageal cancers, some Hodgkin and other lymphomas, seminomas, and chordomas.^25^ There is also growing interest in using PBT to treat prostate cancer, head and neck cancers, and breast cancer. Critics of PBT point to a lack of randomized data supporting its use among these diagnoses. In order to rigorously study the potential benefits of PBT, clinical trials are necessary and ongoing. The presence of travel-related barriers may prevent successful trial completion or lead to results that inadequately represent the general population due to selection bias toward those able to overcome travel-related hardships. That said, our finding of lower drive times to PBT facilities for Hispanic, non-Hispanic Asian, and non-Hispanic Black individuals in the US presents an opportunity to enhance clinical trial enrollment for underrepresented racial and ethnic minority groups.

It is critical to recognize that drive time to a PBT facility is one of many factors associated with realized health care accessibility. Differences in health care literacy that correlate with education may affect a patients’ propensity to seek treatment regardless of geographic accessibility.^26^ Disparities in patients’ ability to pay for treatment due to lower income or insurance status may also preclude treatment even when geographically accessible.^27,28,29^ While Black individuals have shorter driving times to PBT facilities, a retrospective analysis of patients with prostate cancer found that the ratio of Black individuals compared with other races receiving PBT was lower than the ratio of Black individuals receiving intensity modulated radiation therapy.^30^ The higher cost of proton therapy and the higher rates of living below the federal poverty line and uninsured persons among Black individuals likely explain this difference.^31^

### Limitations

This study has limitations. Although this study draws strength from a comprehensive data source to explore granular associations at a national scale, it nonetheless is limited by factors inherently related to the study design. This analysis assumed that patients with cancer will travel to the PBT facility with the shortest commute, which may not happen in practice. Additionally, we only analyzed personal vehicular drive time and did not account for travel via flying, public transportation, or nonvehicular modes of transportation. This likely resulted in underestimating travel times for those without personal vehicles in urban areas, as those individuals might rely on public transportation, taxis, ride sharing, or other modes that require greater resources than a personal vehicle trip in terms of both time and money. Due to the absence of certain racial and ethnic demographics in many census tracts, we could only analyze US residents who were Hispanic, non-Hispanic Asian, non-Hispanic Black, and non-Hispanic White. This analysis may also underestimate the geographic access for pediatric patients because we excluded 2 clinics that serve them exclusively. Personal travel times will vary from those assessed using the census tract geographic centroid, with greater discrepancies possible in larger, more sparsely populated tracts.

### Conclusion

In this nationwide cross-sectional study of driving time accessibility to proton therapy facilities, we found that geographic isolation via road travel was associated with certain patient populations, such as older individuals (aged ≥65 years), those living below the federal poverty line, and suburban or rural residents. These results have important implications for disparities in health care delivery and may also affect the successful accrual of clinical trials evaluating the efficacy of proton therapy.

## Notes

Supplement 1. **eTable 1.** List of Currently Operating Proton Beam Therapy Facilities in the United States as of September 1, 2023 **eTable 2.** Driving Time Between Locations for National Cancer Institute (NCI)-Designated Cancer Centers With an Affiliated Proton Beam Therapy (PBT) Facility (n=23) **eTable 3.** Driving Time to the Nearest Proton Beam Therapy (PBT) Facility for National Cancer Institute (NCI)-Designated Cancer Centers Without an Affiliated Facility (n=40) **eFigure.** Driving Time to Nearest Proton Beam Therapy Facility by Population Subgroup Quintiles by (A) Race and Ethnicity, and (B) Social Determinants of Health

Supplement 2.
Data Sharing Statement
