# Supplementary material for: Travel-Time Disparities in Access to Proton Beam Therapy for Cancer Treatment
Source: JAMA Netw Open. 2024 May 17;7(5):e2410670. doi: 10.1001/jamanetworkopen.2024.10670 (PMC11102024; doi:10.1001/jamanetworkopen.2024.10670)
Supplement: Supplement 1. — eTable 1. List of Currently Operating Proton Beam Therapy Facilities in the United States as of September 1, 2023 eTable 2. Driving Time Between Locations for National Cancer Institute (NCI)-Designated Cancer Centers With an Affiliated Proton Beam Therapy (PBT) Facility (n=23) eTable 3. Driving Time to the Nearest Proton Beam Therapy (PBT) Facility for National Cancer Institute (NCI)-Designated Cancer Centers Without an Affiliated Facility (n=40) eFigure. Driving Time to Nearest Proton Beam Therapy Facility by Population Subgroup Quintiles by (A) Race and Ethnicity, and (B) Social Determinants of Health [file jamanetwopen-e2410670-s001.pdf]

Supplemental Online Content

Burus T, VanHelene AD, Rooney MK, et al. Travel-time disparities in access to proton beam therapy for cancer treatment. *JAMA Netw Open.* 2024;7(5):e2410670. doi:10.1001/jamanetworkopen.2024.10670

eTable 1. List of Currently Operating Proton Beam Therapy Facilities in the United States as of September 1, 2023

eTable 2. Driving Time Between Locations for National Cancer Institute (NCI)-Designated Cancer Centers With an Affiliated Proton Beam Therapy (PBT) Facility (n=23)

eTable 3. Driving Time to the Nearest Proton Beam Therapy (PBT) Facility for National Cancer Institute (NCI)-Designated Cancer Centers Without an Affiliated Facility (n=40)

eFigure. Driving Time to Nearest Proton Beam Therapy Facility by Population Subgroup Quintiles by (A) Race and Ethnicity, and (B) Social Determinants of Health

This supplemental material has been provided by the authors to give readers additional information about their work.

© 2024 Burus T et al. *JAMA Network Open.*

eTable 1. List of currently operating proton beam therapy facilities in the United States as of September 1, 2023

Facility	Address
Ackerman Cancer Center Proton Therapy Center	10881 San Jose Boulevard, Jacksonville, FL 32223
California Protons Cancer Therapy Center	9730 Summers Ridge Rd, San Diego, CA 92121
Cincinnati Children's Pediatric Proton Therapy Center / Proton Therapy at University of Cincinnati Medical Center	7777 Yankee Road, Liberty Township, OH 45044
Beaumont Proton Therapy Center	3571 West 13 Mile Road, Royal Oak, MI 48073
David C. Pratt Cancer Center at Mercy Hospital	607 S New Ballas Rd Suite 3300, St. Louis, MO 63141
Dwoskin Proton Therapy Center Sylvester Comprehensive Cancer Center University of Miami Miller School of Medicine	1351 NW 14th Street, Miami, FL 33125
Emory Proton Therapy Center	615 Peachtree Street NE, Atlanta, GA 30308
Fred Hutchinson Cancer Center - Proton Therapy	1570 N 115th St, Seattle, WA 98133
Hampton University Proton Therapy Institute	40 Enterprise Parkway, Hampton, VA 23666
Inova Schar Cancer Institute / Inova Mather Proton Therapy Center	3225 Gallows Road, Falls Church, VA 22031
Maryland Proton Treatment Center	850 West Baltimore Street, Baltimore, MD 21201
James M. Slater, MD Proton Treatment & Research Center at Loma Linda University Cancer Center	11234 Anderson St, Loma Linda, CA 92354
Mass General Protons	55 Fruit Street, Boston, MA 2114
MD Anderson Cancer Center Proton Therapy Center	1840 Old Spanish Trail, Houston, TX 77054
McLaren Proton Therapy Center	4100 Beecher Rd, Flint, MI 48532
MedStar Georgetown University Hospital Proton Therapy Center	3800 Reservoir Rd NW, Washington, DC 20007
Miami Cancer Institute Proton Therapy Center at Baptist Health South Florida	8900 N Kendall Dr, Miami, FL 33176
Northwestern Medicine Proton Center	4455 Weaver Parkway, Warrenville, IL 60555
Oklahoma Proton Center	5901 W. Memorial Road, Oklahoma City, OK 73142
Penn Medicine | Virtua Health Proton Therapy Center	200 Bowman Dr, Voorhees, NJ 8043

© 2024 Burus T et al. *JAMA Network Open.*

Huntsman Cancer Institute at the University of Utah	1950 E Cir of Hope Dr, Salt Lake City, UT 84112
ProCure Proton Treatment Center	103 Cedar Grove Lane, Somerset, NJ 8873
Penn Medicine Lancaster General Health	2102 Harrisburg Pike, Lancaster, PA 17604
Red Frog Proton Therapy Center / St. Judes Children's Research Hospital	262 Danny Thomas Pl, Memphis, TN 38105
Proton Therapy at The University of Kansas Cancer Center	Rainbow Blvd, Kansas City, KS 66103
Proton International at University of Alabama-Birmingham	400 20th Street South, Birmingham, AL 35233
Roberts Proton Therapy Center at the University of Pennsylvania Health	3400 Civic Center Blvd, Philadelphia, PA 19104
S. Lee Kling Center for Proton Therapy Center at the Siteman Cancer Center	224 S. Euclid Ave., St. Louis, MO 63110
South Florida Proton Therapy Institute	5280 Linton Blvd, Delray Beach, FL 33484
Texas Center for Proton Therapy	1501 West Royal Lane, Irving, TX 75063
Tennessee Oncology Proton Center	4588 Carothers Pkwy, Franklin, TN 37067
The Laurie Proton Therapy Center at RWJBarnabas Health	141 French Street, New Brunswick, NJ 8901
Stephenson Cancer Center at Oklahoma University	800 NE 10th St, Oklahoma City, OK 73104
The Johns Hopkins National Proton Center	5255 Loughboro Rd NW, Washington, DC 20016
The Mayo Clinic Proton Beam Therapy Center - Arizona	5777 E. Mayo Boulevard, Phoenix, AZ 85054
The Marjorie & Leonard Williams Center for Proton Therapy at Orlando Health Cancer Institute	1400 S Orange Ave., Orlando, FL 32806
University Hospitals Proton Therapy Center	11100 Euclid Ave., Cleveland, OH 44106
The New York Proton Center	225 East 126th Street, New York, NY 10035
Thompson Proton Center at Covenant Health	6450 Provision Cares Way, Knoxville, TN 37909
The Mayo Clinic Proton Beam Therapy Center - Minnesota	190 2nd St NW, Rochester, MN 55901
Willis-Knighton Cancer Center	2600 Kings Highway, Shreveport, LA 71103
University of Florida Health Proton Therapy Institute	2015 North Jefferson St., Jacksonville, FL 32206

© 2024 Burus T et al. *JAMA Network Open.*

eFigure. Driving time to nearest proton beam therapy facility by population subgroup quintiles by (A) race and ethnicity, and (B) social determinants of health

A

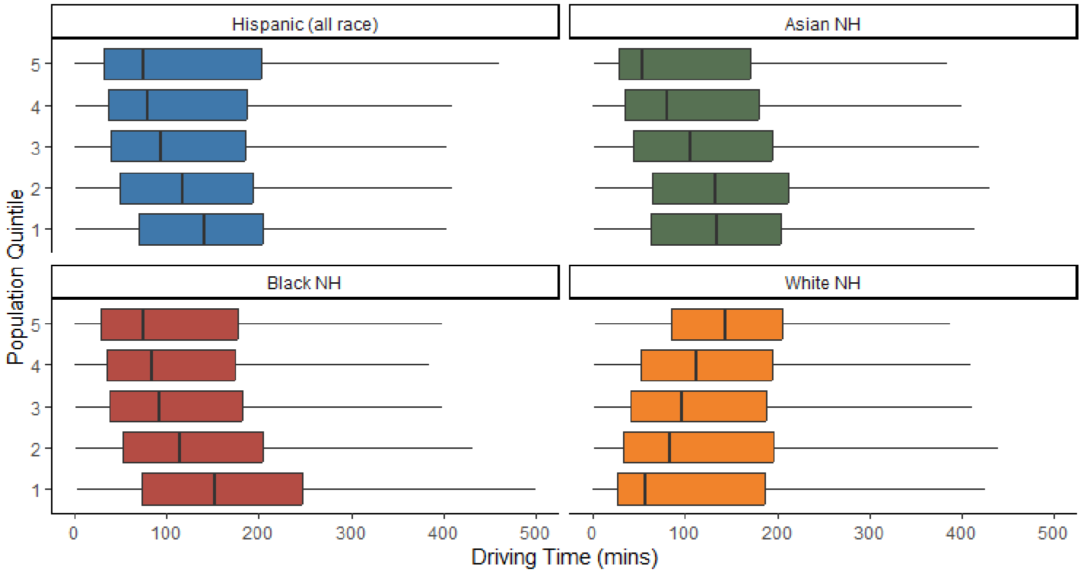

B

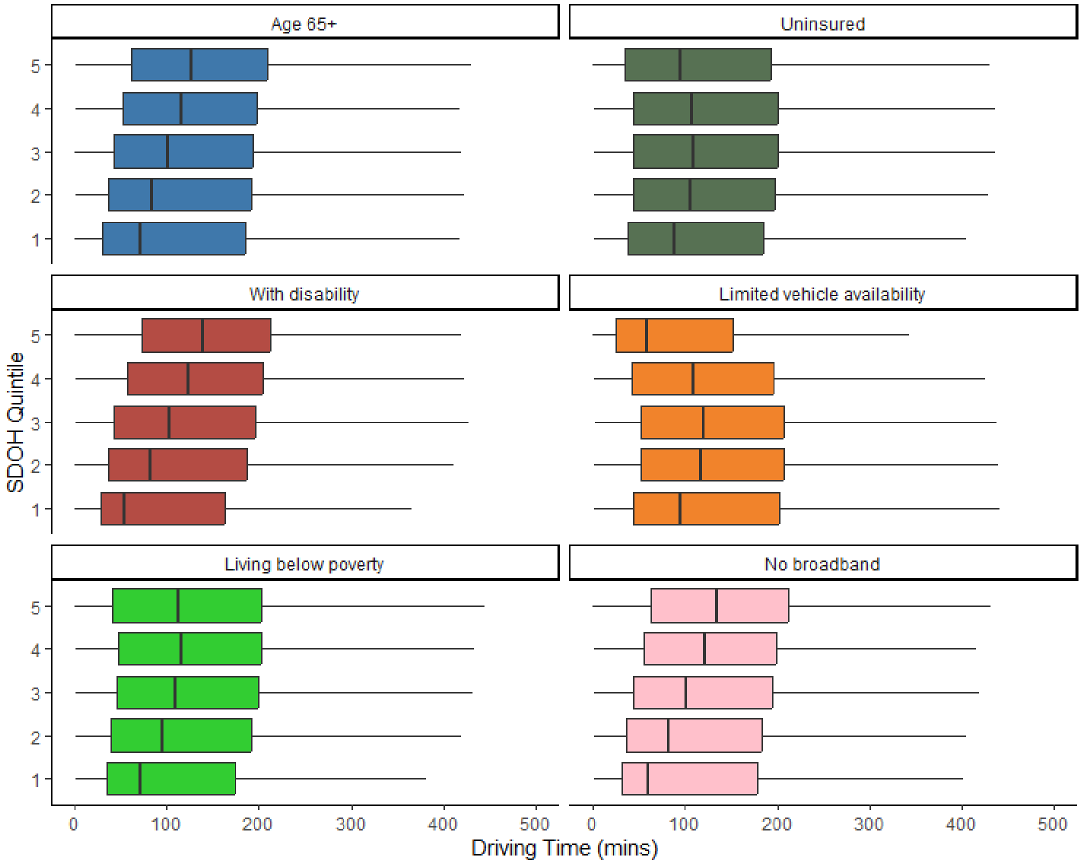

© 2024 Burus T et al. *JAMA Network Open.*

eTable 2. Driving time between locations for National Cancer Institute (NCI)-designated Cancer Centers with an affiliated proton beam therapy (PBT) facility (n=23)

NCI-Designated Cancer Center	PBT facility	Driving time in minutes
Stephenson Cancer Center at University of Oklahoma	Stephenson Cancer Center at Oklahoma University	0.3
University of Kansas Cancer Center	Proton Therapy at The University of Kansas Cancer Center	0.4
Huntsman Cancer Institute at University of Utah	Huntsman Cancer Institute at the University of Utah	0.5
Alvin J. Siteman Cancer Center at Washington University School of Medicine & Barnes Jewish Hospital	S. Lee Kling Center for Proton Therapy Center at the Siteman Cancer Center	0.7
Abramson Cancer Center at University of Pennsylvania	Roberts Proton Therapy Center at the University of Pennsylvania Health	1.6
Sylvester Comprehensive Cancer Center at University of Miami Health Center	Dwoskin Proton Therapy Center Sylvester Comprehensive Cancer Center University of Miami Miller School of Medicine	1.7
Case Comprehensive Cancer Center at Case Western Reserve University	University Hospitals Proton Therapy Center	1.9
Rutgers Cancer Institute of New Jersey at Rutgers Health	The Laurie Proton Therapy Center at RWJBarnabas Health	2.2
Mayo Clinic Comprehensive Cancer Center	The Mayo Clinic Proton Beam Therapy Center - Minnesota	2.3
University of Maryland Marlene & Stewart Greenebaum Comprehensive Cancer Center	Maryland Proton Treatment Center	2.6
Lombardi Comprehensive Cancer Center at Georgetown University	MedStar Georgetown University Hospital Proton Therapy Center	3.4
University of Texas MD Anderson Cancer Center	MD Anderson Cancer Center Proton Therapy Center	4.6
Tisch Cancer Institute at Icahn School of Medicine at Mount Sinai	The New York Proton Center	6.6
Dana-Farber/Harvard Cancer Center	Mass General Protons	10.2
Memorial Sloan-Kettering Cancer Center	The New York Proton Center	11.8
Moores Comprehensive Cancer Center at University of California San Diego	California Protons Cancer Therapy Center	12.1
Fred Hutchinson Cancer Center at University of Washington Cancer Consortium	Fred Hutchinson Cancer Center - Proton Therapy	12.7
Winship Cancer Institute at Emory University	Emory Proton Therapy Center	13.7

© 2024 Burus T et al. *JAMA Network Open.*

Montefiore Einstein Comprehensive Cancer Center (MECC) at Albert Einstein College of Medicine	The New York Proton Center	14.1
O'Neal Comprehensive Cancer Center at University of Alabama at Birmingham	Proton International at University of Alabama-Birmingham	19.3
Robert H. Lurie Comprehensive Cancer Center at Northwestern University	Northwestern Medicine Proton Center	52.3
Sidney Kimmel Comprehensive Cancer at Center at Johns Hopkins University	Johns Hopkins Proton Therapy Center	60.7
University of Florida Health Cancer Center	University of Florida Health Proton Therapy Institute	87.1
**Total**	**Median driving time (IQR)**	**4.6 (1.8,13.7)**

Abbreviations: IQR = Interquartile range

© 2024 Burus T et al. *JAMA Network Open.*

eTable 3. Driving time to the nearest proton beam therapy (PBT) facility for National Cancer Institute (NCI)-designated Cancer Centers without an affiliated facility (n=40)

NCI-Designated Cancer Center	PBT facility	Driving time in minutes
Dan L. Duncan Comprehensive Cancer Center at Baylor College of Medicine	MD Anderson Cancer Center Proton Therapy Center	2.8
Herbert Irving Comprehensive Cancer Center at Columbia University	The New York Proton Center	11.8
Sidney Kimmel Cancer Center at Jefferson Health	Roberts Proton Therapy Center at the University of Pennsylvania Health	12.7
Harold C. Simmons Comprehensive Cancer Center at University of Texas Southwestern Medical Center	Texas Center for Proton Therapy	13.1
Laura & Isaac Perlmutter Cancer at NYU Langone Health	The New York Proton Center	14.3
Barbara Ann Karmanos Cancer Institute at Wayne State University School of Medicine	Beaumont Proton Therapy Center	21.7
Vanderbilt-Ingram Cancer Center	Tennessee Oncology Proton Center	27.9
Fox Chase Cancer Center	Roberts Proton Therapy Center at the University of Pennsylvania Health	33.7
University of Michigan Rogel Cancer Center	Beaumont Proton Therapy Center	44.6
University of Chicago Comprehensive Cancer Center	Northwestern Medicine Proton Center	59.7
City of Hope	James M. Slater, MD Proton Treatment & Research Center at Loma Linda University Cancer Center	60.8
Chao Family Comprehensive Cancer at University of California Irvine	James M. Slater, MD Proton Treatment & Research Center at Loma Linda University Cancer Center	63.1
Massey Comprehensive Cancer Center at Virginia Commonwealth University	Hampton University Proton Therapy Institute	70.2
Norris Comprehensive Cancer Center at University of Southern California	James M. Slater, MD Proton Treatment & Research Center at Loma Linda University Cancer Center	75.2
Masonic Cancer Center at University of Minnesota	The Mayo Clinic Proton Beam Therapy Center - Minnesota	91.7

© 2024 Burus T et al. *JAMA Network Open.*

Moffitt Cancer Center	The Marjorie & Leonard Williams Center for Proton Therapy at Orlando Health Cancer Institute	96.3
Yale Cancer Center at Yale School of Medicine	The New York Proton Center	97.8
Jonsson Comprehensive Cancer Center at University of California at Los Angeles	James M. Slater, MD Proton Treatment & Research Center at Loma Linda University Cancer Center	109.2
University of Arizona Cancer Center	The Mayo Clinic Proton Beam Therapy Center - Arizona	121.9
University of Virginia Comprehensive Cancer Center	Inova Schar Cancer Institute / Inova Mather Proton Therapy Center	126.0
The Ohio State University Comprehensive Cancer Center at James Cancer Hospital & Solove Research Institute	University Hospitals Proton Therapy Center	138.9
University of Wisconsin Carbone Cancer Center	Northwestern Medicine Proton Center	139.1
UPMC Hillman Cancer Center	University Hospitals Proton Therapy Center	143.0
Dartmouth Cancer Center at Dartmouth Health	Mass General Protons	146.9
Fred & Pamela Buffett Cancer Center at Nebraska Medicine & the University of Nebraska Medical Center	Proton Therapy at The University of Kansas Cancer Center	174.2
Markey Cancer Center at University of Kentucky	Thompson Proton Center at Covenant Health	175.9
Holden Comprehensive Cancer Center at University of Iowa	Northwestern Medicine Proton Center	178.7
Roswell Park Comprehensive Cancer Center	University Hospitals Proton Therapy Center	188.9
Oregon Health Science University Knight Cancer Institute	Fred Hutchinson Cancer Center - Proton Therapy	191.5
Mays Cancer Center at UT Health San Antonio MD Anderson Cancer Center	MD Anderson Cancer Center Proton Therapy Center	198.7
Indiana University Melvin & Bren Simon Comprehensive Cancer Center	Northwestern Medicine Proton Center	200.2
Duke Cancer Institute	Hampton University Proton Therapy Institute	203.3
Lineberger Comprehensive Cancer Center at University of North Carolina Chapel Hill	Hampton University Proton Therapy Institute	219.0
Hollings Cancer Center at Medical University of South Carolina	University of Florida Health Proton Therapy Institute	228.4

Atrium Health Wake Forest Baptist Comprehensive Cancer Center	Thompson Proton Center at Covenant Health	252.9
University of New Mexico Comprehensive Cancer Center	The Mayo Clinic Proton Beam Therapy Center - Arizona	390.7
Stanford Cancer Institute at Stanford University	James M. Slater, MD Proton Treatment & Research Center at Loma Linda University Cancer Center	408.7
University of California at Davis Comprehensive Cancer Center	James M. Slater, MD Proton Treatment & Research Center at Loma Linda University Cancer Center	417.3
Helen Diller Family Comprehensive Cancer Center at University of California at San Francisco	James M. Slater, MD Proton Treatment & Research Center at Loma Linda University Cancer Center	425.4
University of Colorado Cancer Center	Huntsman Cancer Institute at the University of Utah	475.1
**Total**	**Median driving time (IQR)**	**132.5 (60.6,199.1)**

Abbreviations: IQR = Interquartile range

© 2024 Burus T et al. *JAMA Network Open.*
