# Supplementary material for: Travel-Time Disparities in Access to Proton Beam Therapy for Cancer Treatment
Source: JAMA Netw Open. 2024 May 17;7(5):e2410670. doi: 10.1001/jamanetworkopen.2024.10670 (PMC11102024; doi:10.1001/jamanetworkopen.2024.10670)
Supplement: Supplement 2. — Data Sharing Statement [file jamanetwopen-e2410670-s002.pdf]

Data Sharing Statement

Burus. Travel-Time Disparities in Access to Proton Beam Therapy for Cancer Treatment. *JAMA Netw Open*. Published May 09, 2024. doi:10.1001/jamanetworkopen.2024.10670

Data
Data available: No

Additional Information
Explanation for why data not available: All data used in this work is publicly available
